# Vancomycin Spacer-induced Hemolysis

**DOI:** 10.7759/cureus.5848

**Published:** 2019-10-06

**Authors:** Anuraj Sudhakaran, Aparna Baburaj, Kinjal Banerjee, Rajesh Essrani

**Affiliations:** 1 Internal Medicine, Geisinger Medical Center, Danville, USA; 2 Internal Medicine, Geisinger Medicine Center, Danville, USA

**Keywords:** vancomycin induced hemolysis, drug induced hemolysis, spacer induced hemolysis, auto immune hemolytic anemia, drug induced hemolytic anemia, hemolysis after orthopedic procedure

## Abstract

Intravenous vancomycin-induced hemolysis has been documented in the literature. This has been presumed to be due to the development of antibodies against additives that are added along with antibiotics. Herein, we present the case of a 72-year-old male who had hemolysis after the placement of vancomycin spacer, which improved after the spacer was removed.

## Introduction

Drug-induced immune hemolytic anemia (DIIHA) is often a rare and sometimes fatal side effect of some of the medications. These medications include but not limited cephalosporins, penicillin, nonsteroidal anti-inflammatory drugs [1]. DIIHA associated with vancomycin is rare, and there are only very few reported cases of autoimmune hemolytic anemia caused by anti-vancomycin antibodies [2]. Herein, we present the case of autoimmune hemolytic anemia caused due to vancomycin spacer placed during an orthopedic intervention. DIIHA improved after spacer was removed.

## Case presentation

A 72-year-old male presented with a past medical history of type 2 diabetes mellitus, chronic kidney disease stage 3, and atrial fibrillation on warfarin. He underwent a total hip arthroplasty at an outside hospital, which was complicated by a post-operative infection. Per records, he was taken back to the operating room approximately one week after the index surgery. The joint was drained, and he was started on ceftriaxone for two months via a peripherally inserted central catheter (PICC) line, followed by six months of oral cefdinir. Approximately three weeks after the completion of oral antibiotics, he developed pain and swelling in the joint. A joint aspirate demonstrated cloudy brown fluid with 28,700 white blood corpuscles (WBC), strongly suspicious of a septic joint. A two-stage procedure was planned, and he was taken back to surgery, where the prosthesis was removed and replaced with a vancomycin spacer (containing vancomycin of 6 g). The synovial fluid culture was positive for *Enterobacter* and *Enterococcus*. He was started on a planned six-week course of IV cefepime and vancomycin via PICC line. Approximately two weeks into his course of antibiotics, he was readmitted with drop-in hemoglobin from 8.5 to 5.7 and an acute kidney injury (creatinine 3.7 mg/dl from baseline of 1.6 mg/dl). He was found to have a high vancomycin trough level (61.2 micrograms/ml), and IV vancomycin was stopped and replaced with IV daptomycin. His renal function continued to worsen, and hemodialysis was necessary. After initiation of HD, there were biochemical features of ongoing hemolysis and thrombocytopenia, raising concerns for thrombotic thrombocytopenic purpura (TTP) and atypical hemolytic uremic syndrome (aHUS). Renal biopsy, ADAMTS13, and aHUS genetic panels were ordered and showed negative results. He was started on plasmapheresis empirically but was discontinued after two days when ADAMTS13 resulted as normal. Renal biopsy showed acute tubular injury with hemoglobin-positive casts in the setting of chronic changes. He was diagnosed with vancomycin-induced immune hemolytic anemia. He underwent surgical removal of the vancomycin spacer. After the removal of the spacer, his hemoglobin stabilized, and hemolytic parameters significantly improved. Although his renal function has not improved three months post-discharge, his hemoglobin, haptoglobin, and lactate dehydrogenase levels remained within normal limits.

## Discussion

Drug-induced immune thrombocytopenia anemia rarely occurs one in million. There is a sudden decrease in hemoglobin levels after the patients are started on the presumed drug. The most common drugs associated with immune hemolytic anemia include cefotetan, ceftriaxone, and piperacillin [3]. Two postulates explain the interaction of these drugs leading to immune-mediated hemolysis. These drugs may alter the antigens on the red cell, resulting in the production of antibodies that cross-react with unaltered antigen, or the drugs may associate with structures on the red cell and be part of the antigen resulting in haptenic reaction [4]. Higher doses of penicillin cause hemolysis due to the formation of immune complexes [5]. Vancomycin-induced hemolytic anemia has been reported recently. Vancomycin antibodies were detected in these cases. Very few reported cases of DIIHA associated with anti-vancomycin are currently available. In this case, hemolysis continued despite discontinuing IV vancomycin. Only after removing his vancomycin spacer did his hemolytic parameters improve.

Polymethylmethacrylate cement preloaded with antibiotics are used in some cases for prophylaxis and also for the surgical revision of prosthetic infections. These spacers help deliver a high concentration of antibiotics around the joint, which would have been difficult to achieve without significant systemic side effects [6]. Antibiotic concentration in serum was very low after the implantation of the spacer. In the study by Gniadek et al., vancomycin antibodies developed after starting patients on IV vancomycin led to hemolysis. As red blood cells (RBC) pretreated with vancomycin did not react with antibodies, Gniadek hypothesized that vancomycin does not covalently bind to the RBC surface leading to hemolysis. We believe that our patient developed anti-vancomycin antibodies during his initial intravenous therapy resulting in hemolysis. His hemolysis continued even after IV vancomycin was discontinued. The presence of vancomycin-impregnated spacers may have been the source of continued antibody-mediated hemolysis. His hemolytic parameters continued to improve and returned to normal after vancomycin spacer was removed. The cost for vancomycin antibody testing was prohibitive in our case and could not be done. The cessation of hemolysis and the lack of an alternative cause for hemolysis during the time period makes vancomycin-impregnated spacer to be responsible for his continued hemolysis.

## Conclusions

Vancomycin-induced immune hemolytic anemia is a rare cause of hemolytic anemia. Patients with vancomycin-impregnated spacers can have continued hemolysis despite stopping IV vancomycin. There should be a high index of suspicion for continued antibody production in the presence of vancomycin spacers and should be promptly removed. Vancomycin antibody testing should be done if possible before removal.
